# Towards the definition of metrics for the assessment of operational design domains

**DOI:** 10.12688/openreseurope.16036.1

**Published:** 2023-09-11

**Authors:** Bernhard Kaiser, Hendrik Weber, Johannes Hiller, Benjamin Engel

**Affiliations:** 1 ANSYS GmbH, Berlin, Germany; 2 fka GmbH, Aachen, Germany; 3 Institute for Automotive Engineering (ika), RWTH Aachen University, Aachen, Germany; 4 ASAM e.V., Hoehenkirchen, Germany

**Keywords:** Automated Driving, Operational Design Domain, Scenario-based testing

## Abstract

**Background:** The Operational Design Domain (ODD) of an automated driving function defines on which roads and under which environmental conditions the function is safe to operate. It plays an important role in definition, safety analysis and validation of automated driving. In many cases, users want to determine metrics about ODDs, or about ODDs in combination with other work products, like collections of validation scenarios. Such metrics could answer questions such as what percentage of the road network of a given region is inside the ODD. While language formats to specify ODDs have emerged over the last few years, a solid methodology on how to calculate different sorts of metrics is still ONThe roadmap for the future.

**Methods:** This contribution suggests metrics for ODDs that are mathematically built upon a notion of ontologies, and ODDs as multi-dimensional cross-products of sets, using standard arithmetic and set operations. To illustrate the idea, a couple of possible metrics for ODDs are derived as examples and discussed in the light of some real-world use cases.

**Results:** To illustrate the application of a ODD metric, we apply an analysis of a sample trip and calculate the theoretical availability of variants of an automated driving system with different ODDs.

**Conclusions:** The metrics presented and the shown sample application present an important next step in discussions around ODDs of Automated Driving Systems. They make it possible to not only consider an ODD specification as a reference for a single system, but allow comparing systems with different ODDs, judging the maturity of a system with a certain ODD, or provide indicators how usable a system is within a real-word application.

## Introduction

The specification of the Operational Design Domain (ODD) is a fundamental step when developing and validating an automated driving system (ADS) or advanced driver assistance system (ADAS) for road vehicles ( 1– 4). Application of the idea of ODDs to other domains (e.g. unmanned aerial vehicles, automated trains or ships) are under discussion (e.g. 5) The ODD defines the “specific conditions under which a given driving automation system is designed to function” ^1^ (similar compatible definitions in 3, 6, 7). A clear specification of the ODD is key to the safety of ADS, because it defines the capabilities and limitations of an ADS that determine where, when and under which side conditions the ADS can operate safely and as intended. Not only is this information important to end users and regulatory authorities, it is even more relevant for developers, safety analysts and engineers performing validation of such systems by simulation and testing. While end users can understand an ODD definition best in a simplified natural language format, engineers need a more exact, in the best case formally underpinned definition of the ODD of the systems they are developing. Standardization initiatives like ASAM OpenODD ^8^, which is managed by the Association for Standardization of Automation and Measuring Systems (ASAM), have emerged to provide such formally underpinned and widely accepted notations to specify ODDs for ADS. Formal ODD definitions are based on ontologies, which go beyond glossaries defining terms to be used by also modelling how different terms are categorized and relate to each other. Also for ontologies, formal notations are emerging, e.g. ASAM OpenXOntology ^9^. On this background, an ODD definition can be interpreted as a sort of partitioning of the operational domain ontology into one subspace where the ADS is expected to work safely (the ODD) and one subspace where this is not the case.

Many practical use cases related to ODDs also require metrics about ODDs, i.e. about some properties of one single ODD, or about the relations between several ODDs, or about the relation of an ODD to related things, e.g. a set of test scenarios. By metric we understand in this context a query about such relations or properties that renders a number or a rating on an ordinal scale (a qualitative and ordered scale, as in high, medium, low) ^8^. However, as formal notations for ODDs are just emerging, there are no published or commonly accepted definitions or examples for metrics at present.

This article contributes to the establishment of metrics about ODDs by interpreting ODD definitions according to 8 in the light of set theory, using commonly accepted arithmetic and set operations like cardinality of a set or union and intersection of sets to derive a variety of ODD metrics. A few examples for such metrics are given, and their usage is put in the context of typical use cases throughout the development, validation and safety assessment of ADS.

The rest of the article is structured as follows: In Section ’Preliminaries’, we present some preliminaries about ontologies, ODD definition, use cases of ODDs in the development and validation workflow and for metrics. In Section ’A set-theoretic approach to ODD specifications’, we establish our view on ontologies and ODDs based on set theory. In Section ’Deriving methods for ODDs’ we show how ODD metrics can be derived from the concepts introduced before, applying standard mathematical operations. In Section ’A use case example for ODD metrics’ we give some examples how metrics according to our proposals could look like in realistic ADS development workflows. In Section ’Conclusions’ we conclude the paper and give an outlook on standardization and further improvement.

## Preliminaries

### ODD specification and the underlying ontology

In the workflows for development, safety assessment and validation of ADS the ODD specification plays a central role as it serves as an input document for many different purposes ^1, 10, 11^, including requirements elicitation for the ADS (e.g. regarding sensor set and regarding functions and algorithms), hazard analysis for Safety of the intended functionality (SOTIF) acc. ^1^, derivation of validation scenarios and also the design of onboard functions that detect in realtime whether the vehicle is inside the ODD and at which point the ODD is left and appropriate action must be taken (like prompting the driver to take back control). According to the more verbose definition in 6 (as compared to 1), the ODD is the set of “operating conditions under which a given driving automation system or feature thereof is specifically designed to function, including, but not limited to, environmental, geographical, and time-of-day restrictions, and/or the requisite presence or absence of certain traffic or roadway characteristics.” The main topics to be addressed in an ODD specification are the allowed or forbidden road types (e.g. only allowed on highways) and the environmental conditions, with focus on weather conditions including their effects on visibility (e.g. fog) and road surface properties (e.g. reduced friction on snow). Additional specific properties may be of relevance, for instance number and width of lanes, the existence of hard separation between opposite directions of traffic or the presence or absence of certain classes of traffic participants.

All of this can be specified informally in any textual, tabular or graphical format understandable to domain engineers. Some examples can be found in Annex A of 7. Up to this point, neither a formal grammar for the language nor an underlying ontology are required. However, to make machine reasoning on ODDs possible (e.g. checks whether a driving scenario lies entirely inside the ODD) a more formalized machine-readable Domain-Specific language (DSL) is required. Proposals for such formalized languages for ontologies and ODDs are known from 12, which influenced our work, and others. To define a formalized DSL for any problem domain, it takes a grammar that defines the language syntax rules and an ontology, or at least a hierarchical taxonomy, that defines the domain-specific terminology. The ontology not only defines the allowed terms (names of object types of relevance) as a glossary would do and their hierarchical classification as a taxonomy would do but also defines attributes and their allowed value ranges for each object type and puts the different objects in relation to each other. Essential to have are (sub)classification relations between object classes and categories, e.g. car, bus, truck and motorcycle are all subclasses of motor vehicle, which is in turn a subclass of vehicle, or composition relations, e.g. a country road can have 1 to 4 lanes. In addition to what a taxonomy can offer an ontology provides relations like: car is a sub class of vehicle and vehicles drive on driving grounds and road is a sub class of driving ground. Expressions on individual instances like „Ego car is driving on highway A100” can be formulated during scenario specification and can then be checked for validity against the ontology. The ontology is a central pillar of the ADS engineering process because it serves as a formal underlying agreement on terms for requirements specification, ODD specification, safety analysis, test scenarios and more. While we build our theory on ODD metrics on an ontology, we can state that a hierarchical taxonomy with the option to define attributes for different kinds of objects is the minimum requirement to define ODD metrics as proposed by this paper.

ASAM OpenXOntology ^1^ ^9^ has been proposed as basic framework for specifying ontologies within the domain of ADS by providing a common basic architecture, root classes and elements that are frequently used (like road, vehicle etc.) using the Web Ontology Langauge (OWL) ^13^ and the Semantic Web Rule Language (SWLR) ^14^ for implementing the ontology and rules. It makes sense to standardize not only the formal language for ontologies, but also some reference ontologies for different kinds of vehicles and application domains, because working on a common ontology enables exchange of ODD and scenario specifications based on this ontology between different parties like carmakers and sensor suppliers. The ontology also serves as a sort of checklist of relevant aspects for the ADS and therefore helps improving the quality of an ODD specification. 7, 15 and 16) make proposals of items which should be considered in an ODD definition and accordingly should appear in the underlying ontology. Once the ontology has been specified in a common ontology syntax or dedicated DSL, the specification of the ODD basically consists in declaring which objects and constellations outlined by the ontology are inside the ODD and which of them are not. For instance, if the ontology defines road type to be one of (highway, country road, city street), we could state for a given AD function that it can operate on highway but not on country road and city street. Reality is more complicated, not just because the ontology contains many details that relate more with the dynamic aspects of scenarios (driving maneuvers etc, see 9) which are not relevant for an ODD specification, but also because we would have to make an in/out decision for each combination of attributes from the ontology (e.g. highway and 3 lanes and rainy weather and dense traffic etc.). One common approach to simplify things is to accept an incomplete (with respect to the ontology) specification and add a meta-statement about the ODD specification to be permissive (everything that is not explicitly mentioned is allowed) or restrictive (everything that is not mentioned is forbidden) ^8^. A practicable DSL for ODDs also needs to be able to cope with the fact that an ODD is not always a contiguous and regularly shaped space, which may be due to conditional restrictions (e.g. highway usage is allowed at day and night, but country road usage only at daytime) or unconnected sections (e.g. a lane keeping function works at 60 kph or more on a highway, at speed up to 50 kph in a city, and not at all on a country road), and it can contain exceptions (e.g. a traffic jam pilot can operate well on highways, but not in construction sites). ASAM OpenODD ^8^ is an attempt to provide a standardized ODD specification language that considers all of these requirements, driven by major players from academia, carmakers, tool suppliers and regulation authorities, although at the time of writing this article still in its concept stage.

For the remainder of this paper we assume that the ODD is defined in a formalized notation, as will be OpenODD; the concrete syntax, however, as well as the actual specification is irrelevant for our considerations in the following sections. We will further assume from now that all different ODD specifications we are using and measuring are based on the same ontology or at least on compatible ontologies (discussing the exact meaning of compatibility is beyond the scope of this article).

### ODD metrics

Specification and usage of ODD specifications is a qualitative act (what is in or out?) and does not require any sort of metrics. But there exist many use cases in which users may additionally want to obtain some sort of numerical information about ODDs, and formalized ODD specifications enable the calculation of certain metrics on ODD as we will show. A trivial example is "How big is my ODD?". It is intuitively clear that an ADS that can operate on highways and on country roads in a speed range up to 80 kph has a "bigger" ODD than another ADS that can operate only on highways an only in a speed range up to 60 kph. But it is less clear how the "size" of an ODD can be put in numbers, and how the sizes of the ODDs of two different ADS can be compared in a meaningful manner. Other metrics on ODDs could be related to quality or completeness of an ODD specification, which are important criteria when it comes to justifying trust in automated vehicles and quality management in general. More relevant metrics in practice can be obtained when additional information, in particular statistical data are combined with the plain ODD specification. For instance, (relative) sojourn probability in certain situations, on certain road types or under certain weather conditions (e.g. highway is 20 % of all driving time, or of all km driven), or occurrence frequencies of some (exceptional) events like a pedestrian on a highway are very relevant as an input for risk evaluation or for weighting of test scenarios in a large scenario set. Mapping to an ordinal scale, such as the four "exposure" ratings E1, E2, E3, E4 from ISO 26262 ( 17) makes such statistical exposure information available to hazard analysis according 17 and 1.

Many useful metrics related to ODDs are not just about ODDs, but put ODDs in relation to other sorts of things, in particular scenarios or sets of scenarios. Of particular interest are coverage metrics, which can be used to assess how densely and completely a test scenario covers a given ODD, ultimately leading to the all-important decision: "When have we sufficiently tested the ODD?". Just as there are many different coverage metrics in software testing ^18, 19^ there may also be more than just one possible metric for ODD coverage. Other metrics relating a scenario to an ODD concern the question what percentage of a certain ride (e.g. a typical daily commute to work) lies inside the ODD where the driver can turn the automation on, or how often on a certain scenario "ODD gaps" occur so that the driver needs to take back manual control - all important buying decisions for cars equipped with ADS.

The OpenODD concept ^8^ presents more possible applications for metrics and suggests also some candidate metrics, which were only informally defined as items for further discussion. Each of them is motivated by a user story like "As an ADAS/AD designer, I want to measure the size and restrictiveness of my ODD to see how broad the usage scope of my ADAS is." Candidate metrics for this use case could be the weighted attribute values or value ranges that are forbidden by an ODD spec w.r.t. the ranges defined by the underlying ontology or, as a more specific example, what percentage of all road kilometers in Germany are suitable for a given automation feature according to its ODD specification (in general, or related to specific attributes of the ODD like road type, weather etc.)? According to the intuitive introduction given in 8, a metric is a function that maps some property about one or about several related things onto an ordered scale (e.g., a point scheme reaching from 1 to 10 points). Metrics allow propositions about the quality, completeness, work progress etc. related to ODD specification or ODD usage (e.g. to derive vehicle requirements, to specify test or simulation scenarios).

## A set-theoretic approach to odd specifications

For our approach to defining some useful ODD metrics as conceptualised in 8, we now interpret the ontology underlying ODD as a tuple of sets, either discrete or continuous sets, depending ONThe attribute we are talking about. This is most natural for object classes and attributes in the ontology that allow only the selection of one value from a given enumerated list. For instance, let there be an object named road and let it have an attribute "Road Type", for which you can select from the list "Highway", "Country Road" and "City Street". Now let’s interpret this as a set. By convention, we will use for such sets italic typesetting, a capital initial letter and a plural-s in the end, e.g. *RoadTypes*. We define the set *RoadTypes* by explicit enumeration of its elements, e.g. *RoadTypes* = *{Highway, CountryRoad, CityStreet}*

In some cases the ODD aspect is represented by a natural number. In this case, the set where this data item can be mapped onto is obviously the set of natural numbers, or a subset thereof. A typical example would be the number of lanes of a given road.

Finally, attributes can deal with things we can measure, for instance lane width, vehicle mass or rain intensity in millimeters per square meter per hour. While it makes sense for an ontology representation language to be able to handle different physical dimensions and values with units, it is obvious that all of these attribute ranges can be mapped, after removing the unit, onto the set ℛ of real numbers or a subset thereof. In many cases domain engineers may want to restrict the range of allowed values as appropriate for the purpose, e.g. the range for "lane width" could be restricted to the interval [2.0 m .. 4.0 m] if other lane widths cannot legally exist in a given country.

The entire ontology is then represented by a multi-dimensional hyperspace, spanned by the cross-product of the sets for each aspect.

Example:

- *RoadTypes* = { *Highway*, *CountryRoad*, *CityStreet*}
- *Weathers* = { *clearsky, cloudy, rain, snow, fog*}
- *NumberOfLanes* ∈ ℕ
- *LaneWidths* = [2.0 *m* . . . 4.0 *m*]

Accordingly, if we denote the set-theoretic representation of our ontology as *ONT*, then


ONT=Roadtypes×Weathers×NumberOfLanes×LaneWidths

In realistic cases, ontologies can be more complex than this. For instance, there may be hierarchies of classes (e.g. roads are first classified into "inside city" and "outside city", then "outside city" roads are further subclassified into highway or country roads), or there can be categories with multiple sub categories (e.g. weather can have sub categories wind, precipitation, visibility, each one coming with their specific attribute values). In this situation our approach can still be applied by flattening the hierarchy.

Our approach of expressing an ontology as a cross-product of sets is still over-simplistic as it only lists objects and their attribute ranges, but not relations. However, for our purpose of ODD metrics this is sufficient in most cases; relations are rather important when dealing with scenarios defined by means of the ontology (e.g. There is a relation "is driving on" between members of *Cars* and members of *Roads*, so that we can express that "car X is driving on road y"). In case we actually need relations, they can be expressed as tuples selected from the cross-product of two sets, so we could extend our formalism easily. In the standard case, we just abstract unnecessary details away when mapping the ontology definition given in any suitable language onto sets.

After the ontology we can now equally specify the ODD in terms of set theory. If we say that the ODD is the subset of the "driving universe" which is safe for driving in automated mode, then we get *ODD* ⊂ *ONT*. More specifically, we can form component-wise subsets for each aspect (each dimension of the vector of sets) of the ontology *ONT*.

Let us assume for instance that for some given ADS, the ODD is verbally characterized as follows:

•   Only highway and country road is allowed, but not city

•   Any weather except fog is allowed

•   Two or more lanes are required

•   Lane width must be at least 2.5 m

Then, accordingly, if we denote the set-theoretic representation of our ODD as *ODD*, we can state that


ODD={highway,countryroad}×Weathers\{fog}×{n|n≥2} ×[2.5,4.0]

which is a subset of the ontology.

Also more complex ODDs with conditional limitations can be expressed as sets, e.g. the previous example "highway usage is allowed at day and night, but country road usage only at daytime" could be expressed as


ODD={highway}×Weathers∪{highway,countryroad}×{sunny}

Using set theory to define our ontology and ODD gives us access to a well-established toolbox of operations, like set union or intersection, and to measures to determine the "size" of a set.

## Deriving metrics for odds

### Some initial examples

According to the definition in 8 a metric ^2^ is "a function that transforms features or properties of an ODD – alone or in its relation to other ODDs, to scenarios etc. – into a number or an ordered set of qualifiers (e.g. good > medium > bad)."

This means, a simple (but not necessarily useful) metric on the example ODD given above is already given by the cardinality, i.e. the number of elements in an explicitly enumerated set describing one ontology attribute. For instance, our ADS can operate on highways and on country roads, which means that it can operate on 2 road types. Two is a number, so this is a metric. A little more helpful might be the knowledge about what fraction of the existing (known, or commonly defined) road types from the ontology this ADS can cover. Our example ontology contains 3 different road types (highway, country road, city street), of which 2 are inside the ODD. So we can say that our ADS is operable on two thirds or 66.7 % of all existing road types. This is a slightly more advanced metric. These metrics can already be used to compare different ADS (e.g. serving a similar purpose, but from different vendors) by their ODD, e.g. a system that can operate on 2 out of 3 road types is in some regard superior to another system that can operate only on 1 out of 3 road types.

### Weighting of the values per attribute

The primitive metrics proposed until now are still not at a level to answer ODD stakeholders’ questions. To evaluate the market prospects of an ADS, it is more important to know on what percentage of all roads (not: road types) in a given market region (say, the European Union) the vehicle can operate in automated mode. To achieve measures for that purpose, we can apply weighting to the attribute values, e.g. derived from available statistics. Let us assume for example that in a relevant market region we have the following statistical distribution of the three allowed road types:

•   Highway: 25% of all road kilometers

•   Country road: 40% of all road kilometers

•   City street: 35% of all road kilometers

The question of interest is here: On what portion of all road kilometers can my car operate with ADAS function switched on (based only on road types, for now ignoring weather and further aspects constrained by the ODD). The answer is: 65%, because our ADS can operate on highways (25% of all road kilometers) and on country roads (40% of all road kilometers), and in the given setting it makes sense to add up the weights for the values that belong to the ODD. Note that in this setting the weights of all road types defined by the ontology must sum up to 100%.

Of course, the selection of weighting factors is up to the user and can be driven by their specific business needs. For instance, instead of weighting the road types by their statistical fraction over a whole country (there may be many kilometers of road that are seldom used) we could rather look at the typical daily commute of an average employee and take the weights from the portions of road types he is riding on.

An analog approach as for explicitly enumerated sets can also be directly applied if the attribute from the ontology is mapped onto a finite subset of the natural numbers. It is more difficult for infinite discrete (countable) sets, in particular the entire set of the natural numbers. For instance, an ontology attribute like "numbers of lanes" of a road could be mapped to the set ℕ of natural numbers (which is a likely choice), and the ODD of our ADS may specify that we can operate on any road with 3 or more lanes. Now we cannot simply divide the number of possibilities included in the ODD by the total number of possibilities, because both numbers are infinite. However, this is not much of a restriction in practice: As a simple remedy we can define a reasonable upper bound (e.g. ten lanes), or we can refer to other metrics that are not affected by this issue. E.g. even if we allow an unlimited number of lanes the weighting by occurrence probabilities for different numbers of lanes would still work, but all numbers greater than 4 would have low or even zero occurrence probability.

### Continuous value ranges

We have seen that there are also attributes that are defined over a continuum of values (e.g. lane width, visibility range, rain density). Here we would run into difficulties with our counting approach, because ℝ is an uncountable set. The mathematical cardinality does not help us here because all sub sets of R have the same cardinality. However, intuitively we might say that if the allowed range of lane widths specified by the ontology is from 2.0 m to 4.0 m, and our ADS can operate on all roads with a width of 2.5 m or wider (i.e. on the range [2.5, 4.0]), then it covers 75% of the range. What we have done here is to define the "size" of a one-dimensional, continuous range of values as max_value - min_value, and then optionally set such sizes into relations to the whole range allowed by the ODD, just as we did for countable value ranges. If the domain defined by the ontology goes to infinity (the range of weather-dependent optical vision could be such an example), then in most practical cases it is possible to arbitrarily define a meaningful upper limit of relevance (e.g., a visibility of 1000 m could be considered an upper limit of attribute values). We could also specify a discrete symbol for all values that are above a certain range, e.g. *Visibilities* = [0.0 *m*, 1000.0 *m*] ∪ ” *clear*”, where "clear" as a discrete value gets a fraction of the probability and the rest is defined by a probability distribution over the continuous range from 0.0 to 1000.0 m. If the allowed (by the ODD) range is not contiguous (e.g. 2.0 to 3.0 m and then again 4.0 to 6.0 m are valid ranges for lane width then the individual "sizes" of the subareas can be added up. In the same way as for discrete value sets, weighting by statistical probability densities is also possible. To do so we would have to form the integral over the probability densities on the value range (e.g. the lane widths of all roads in some country could be distributed according to a truncated Gaussian normal distribution). This will give a weighted metric for the range covered by the ODD that will serve the purpose in many practical cases.

An alternative solution (that often needs to be applied anyway for later testing and simulation purposes) is to divide the continuous range into equivalence classes (equidistant or not, according to the purpose), e.g. visibility range < 50 m, 50.. 100 m, 100 .. 200 m, 200 .. 1000 m, > 1000 m. Then all approaches explained for finite sets can be applied.

As an aside, to keep language simple, we will from now mean by saying "size of a set" (denoted as | *S*|):

•   for a finite set the cardinality (number of elements)

•   for a continuous value set the accumulated interval length (max-min) divided by the total range delimited by the ontology.

### From attribute-level metrics to ODD-level metrics

What we’ve done so far has been defining metrics on single aspects (or "dimensions") of the ODD, such as road type or weather. In some cases it may be sufficient or even preferable to discuss and measure the ODD size and compliance based on individual attributes (e.g., what percentage of all typical driving tasks can be performed in automated mode, depending on road type / depending on weather conditions / depending on required V2X infrastructure, etc.). In some cases this can even give better explanations why automated operation is prohibited in some cases, and where to improve in order to make the applicable usage area bigger. However, in some cases, one may want to measure the properties of the ODD as a whole or compare multiple ODDs in their entirety. In these cases, we need to find a way to obtain a single number that serves as "the metric". One way to solve this problem is to smartly combine individual aspect metrics, for example, using linear weighting.

Assume for instance that a given ADS can handle 75% of all roads in a country in terms of lane width and 50% of all roads in terms of road types. Let’s further assume that restrictions caused by insufficient lane width are more important to the customer than road-type related restrictions, so we decide to give this influence factor a weight of 0.8, whereas road type restrictions are considered less important, so we‘ll give them a weight of 0.2 (it is good practice, but not always a requirement, that the weight factors sum up to 1.0). Then a combined rating of 0.7 can be calculated by 0.75*0.8 + 0.5*0.2.

In the general case, we can express this as the scalar product of the vector of individual metrics and a weight factor vector:


$$m=W\cdot D=\sum \;(w_{i}*d_{i})$$

where m is the resulting (scalar) metric, W with its components *w _i_* is the vector of weights and D with its components *d _i_* is the vector of the individual metrics per dimension.

Attention must be paid when applying statistical weighting to individual values per ODD aspect: We can not always expect that these are stochastically independent from each other. For instance, we can say that 30% of all driving statistically occurs on highways and the remaining 70% on other roads, and that 80% of all driving occurs at daytime and 20% at night. But this does not necessarily mean that 30% * 20%, i.e. 6% of all driving is nighttime driving on highways, because it could well be the case that many people tend to avoid highway at night, so that finally only 3% of all driving is driving at night on a highway. This means that both aspects are correlated and we would have to determine a joint probability distribution. Although this complicates things it is not an impossible thing to do and requires nothing but established mathematical principles.

### Comparing ODD attribute-wise by the Jaccard index

A class of more complex and interesting metrics for comparing ODDs that can be obtained by applying the aforementioned principles are inspired by the Jaccard Index, also referred to as Intersection-over-Union (IoU). The IoU is a very common metric to measure the performance of image classification algorithms and works by comparing the dimensions of the reported or predicted bounding box of some figure (e.g. a traffic sign to be recognized on a photo of a street scenery) to the dimensions of the corresponding ground truth image. A sufficiently accurate introduction to the IoU or Jaccard Index can be found in many text books on image classification or on common resources like Wikipedia ^3^. The Jaccard Index computes the similarity of two rectangular areas (usually the ground truth bounding box and the detected bounding box of some object) in terms of size, position and shape as a real number between 0.0 and 1.0, where 1.0 means that the predicted and true bounding boxes are perfectly equal and at the same position, and 0.0 means that they do not overlap at all.

The Jaccard Index is defined as the quotient of the size (acc. to the definition given above) of the intersection of both bounding boxes A and B, and the size of the union of both bounding boxes:


$$IoU=\frac{\left|A\;\cap \;B\right|}{\left|A\;\cup \;B\;\right|}$$

First the intersection/union of the two rectangles is formed in a geometric fashion (area of the overlapping region in case of the interection, added areas of both rectangles minus area of the overlapping region – which would otherwise be counted twice – in case of the union).), then the size is determined by the surface of the resulting figures amd the division is made between these two scalar numbers. This approach can be transferred from the domain of image classification to the domain of ODD metrics. Obviously we are not dealing with two-dimensional and rectangular areas, but with multi-dimensional and irregularly shaped sets, but based on the basic idea we can develop all the tools we need to determine the size of a set, the union and the intersection of sets. We can now compare two ODDs by "equality" on a scale from 0.0 to 1.0 by calculating the Jaccard Index; when it is 1.0 they are equal, when it is 0.0 they don’t overlap in any dimension (i.e. if ADS1 can operate on highway only and ADS2 on city streets only, but both can operate on lane widths between 2.5 and 3.5 meters, then the overlap is already unequal to zero - this shows that in many practical cases we are more interested in overlap metrics per aspect, then by a total overlap metrics).

A metric for the size (as one single number) of an ODD with respect to a given ontology is *IoU* ( *ODD, ONT*). Keep in mind that most ODD metrics we define refer to a given ontology, and comparisons between two ODDs by using metrics are only possible when both ODDs are defined over the same ontology. The definition of metrics providing comparisons like "ODD1 is 20% bigger than ODD2" is straightforward, but note that it should first be checked whether the smaller ODD is a true subset of the bigger one, to make this metric interpretable in an intuitive sense. As further examples we can define quality or completeness metrics for the ODD specification, that tell us what percentage of all information items found in the ontology are addressed (i.e. explicitly allowed or explicitly forbidden) in the ODD specification. One such metric is the number of aspects (like road type, lane width, visibility, rain intensity...) addressed in the ODD specification divided by the number of aspects defined by the ontology.

Additionally, when specifying *ODD _per_* ⊂ *ONT* as the elements explicitly mentioned as allowed in the ODD specification and *ODD _res_* ⊂ *ONT* as the elements explicitly mentioned as forbidden from the ODD, this allows for the definition of further metrics. Following those definitions, the specification completeness can then be stated as


$$SpecificationCompleteness=\frac{\left|ODD_{per}\;\cup \;\;ODD_{res}\right|}{\left|ONT\;\right|},$$

which would return a fraction as metric.

Additionally, all elements not explicitly defined or restricted so far, could be found using


$$missing=ONT\;\backslash \;(ODD_{per}\;\cup \;\;ODD_{res\cdot })$$

### Metrics about ODD coverage by scenarios and on ODD gaps

Finally, an important subclass of ODD metrics are metrics that relate scenarios (in particular: test or simulation scenarios) or entire sets of scenarios to ODDs. A very relevant, but also highly challenging subclass of such metrics are coverage metrics. Note that, similar to software testing, there is not just one definition of coverage. There are different issues when trying to achieve and measure coverage of an ODD, the most obvious one being the huge or infinite space of value combinations allowed by the ODD: even for discrete (or artificially discretized) sets the number of combinations is prohibitively large. If we extend to continuous value ranges (as in lane width) the set of all possibilities is uncountably infinite. Within the multidimensional space spanned by the ODD, a single driving scenario of a vehicle under observation is like an infinitely thin thread, and even many of them will not be able to cover it in a strict sense. Existing methods like forming equivalence classes (e.g. just for degrees of lane width) and reducing combinatorial explosion by approaches like Latin hypercube sampling can render this job feasible (and allow measuring the coverage), but leave many questions open. For instance, often the transitions between different sub-cases are the the ones that provoke safety-critical reactions; this means that it is not enough to show that you drove on a highway in sunlight and in another scenario you drove on a highway in rain, but also that you had a scenarios on a highway where you started in sunshine and later it started to rain. But more importantly, not everything that characterizes a scenario and influences an automated vehicle’s behavior therein appears in the ODD specification - or even the ontology - at all. Further, the ODD mainly focusses on static aspects like road type and geometry, or weather conditions. Regarding traffic participants, it is only menetioned which classes of them can be present, and what speed ranges can be expected for each class. For a test of automated driving functions a lot more details are relevant, like type, shape and even pose or color of traffic participants, the maneuvers they perform, etc. and, similarly, little details about the road surface, vegetation, objects around and so on, which are not even addressed in the ODD specification. And it is questionable whether all those details should be included into the ODD specification to make it "more perfect" because the original intention of an ODD specification was to be a concept-phase document. So the question of (test) coverage metrics is not solely a question of ODD coverage, and therefore lies, at least partially, beyond the scope of this article.

There are, however, other metrics relating scenarios and the ODD, which are more easily implementable and of high relevance for ODD engineers. Such metrics could deal with questions like:

•   Within a given scenario (e.g. a ride from City A to City B), how often does the driver have to take back control because the ODD is temporarily left (ODD gaps)?

•   Which percentage (by kilometers, or by travel time) of a given scenario can be done in automated mode, i.e. is inside the ODD?

•   What is the average length of a time interval where the automation can be constantly turned on over a given scenario?

These metrics are often of relevance for the market prospects of automation functions, because the perceived value of an automation function for the end user is determined by exactly these questions. They can also be used as success metrics in a car maker’s effort to reduce more and more ODD gaps and to extend the usability space of their ADS.

There are various options how to calculate these kind of metrics, of which we can only present a few examples in the scope of this article:

In some cases a Boolean predicate (e.g. "Physical separation between directions of traffic present") can be assigned to each point of the road, as derived by a feature-enriched road map. Then we can query and integrate over the road segment of interest (e.g. highway A9 southbound between junction Leipzig and junction Nuremberg) or the path of a given scenario or use case (e.g. typical daily commute to work) and aggregate the distance where the condition is true. Doing so we can calculate metric like:

•   Percentage of the distance where the condition is true (in this example: percentage of highway with physical separation of directions)

•   Maximum, minimum or average length of segments where the condition is true or false (e.g. longest piece of highway without physical separation along this journey)

•   Number of changes from true to false or from false to true (e.g. how often does it occur that the physical separation ends, which might be a reason to end automated driving and call the human driver to action)

Likewise, we can do with properties that are not Boolean but expressed as numbers (e.g. number of lanes, slope) and with things or events we can count (e.g. how often do we enter e tunnel on this road segment). If the ODD excludes such phenomena (tunnels are not allowed) or requires constraints on them (e.g. must have at least 2 lanes per direction) then this leads directly to ODD metrics.

## A use case example for odd metrics

**Figure 1.**
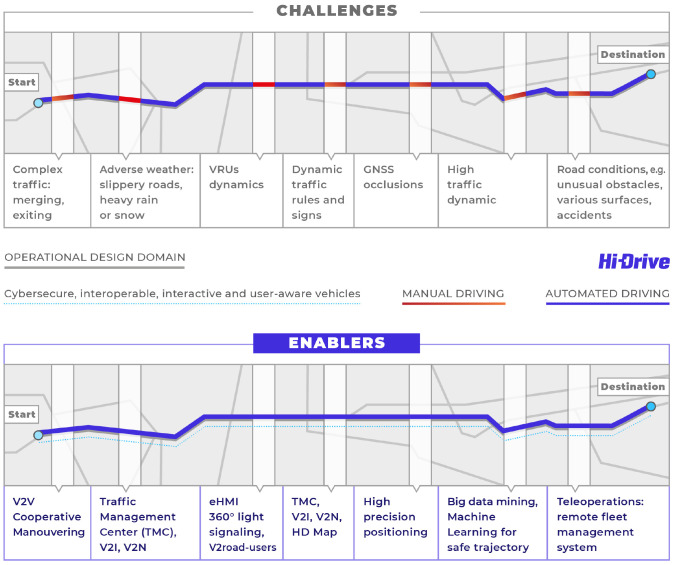
Current SAE L2-L3 automated driving fragmented by limitations in ODD - and eased by Hi-Drive. © Hi-Drive.

The aim of the Hi-Drive research project ^4^ is pushing the state-of-the-art further up towards "High Automation" with the overall goal of improving automated driving. Functions available or in development often have a limited ODD, e.g. limited by geography, visibility or traffic conditions. Hi-Drive strives to extend the ODD and reduce the frequency of take-over requests. This is achieved by selecting and implementing enabling technologies, which bridge these gaps. Some examples such as vehicle-to-infrastructure (V2I) communication are mentioned in Figure 1.

This highlights the need for a clear and structured definition of ODDs, and subsequently also ODD metrics to enable Hi-Drive partners to analyze the gained benefits by these enabling technologies.

**Figure 2.**
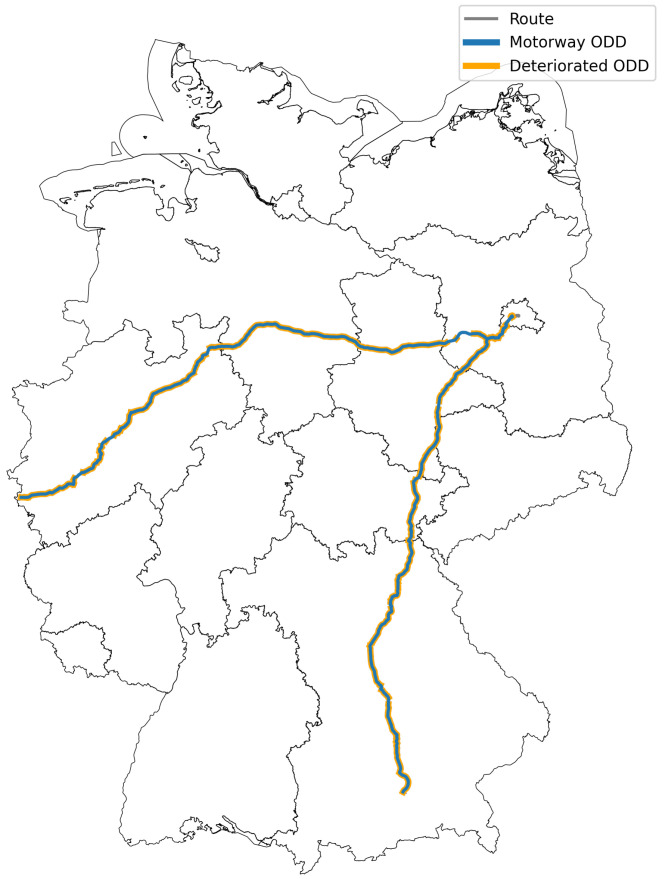
The route analyzed for this paper. Contour Germany: © GeoBasis-DE / BKG 2022.

To get an overview of how such metrics can help analyze the ODD, consider the following example. Using an automated vehicle, a trip from Aachen via Berlin to Höhenkirchen-Siegertsbrunn is planned. Much of the distance covered here is done on motorways with some parts on local and regional roads at the beginning (in Aachen), in Berlin in the middle of the trip and at the end of the trip just south of Munich (see Figure 2).

This means, that most of the trip is on divided motorways, which have at least two lanes per direction. A customer’s expectation would therefore be that the function could be activated in Aachen and the system would have to be deactivated only shortly before Berlin for the first time, as everything in between is motorway. Of course, this would be the most idealistic assumption and would take approx. 11.5 hours for the ride with a maximum travelling speed of 120 km/h.

The automation function is basically intended to be used on motorways with physical separation, so this is the most restricting factor of its ODD. If we assume at first that the vehicle can cover all aspects of a motorway trip, this would result in around 93 % of the trip covered by the ODD time-wise and almost 98 % measured by the distance (see Table 1). We note that, for similar purposes, different metrics can be formulated (fraction of time vs. fraction of distance), which may produce different results.

**Table 1.** T he metrics for the different ODDs along the route.

Metric	"Ideal" ODD	Segmented ODD	Non-Deteriorated ODD
Total Distance (in ODD)	1206.1 km (625.3 km / 580.8 km)	1116.6 km (577.7 km / 538.9 km)	306.3 km (160.9 km / 144.3 km)
Total Duration (in ODD)	10.82 h (5.7 h / 5.1 h)	10.02 h (5.3 h / 4.7 h)	3.0 h (1.65 h / 1.34 h)
Longest Duration without take-over	5.7 h / 5.1 h	601 s / 615 s	max. 5.7 h / max. 5.1 h
Number of take-overs	1 / 1	136 / 122	min. 1 / min. 1

**Figure 3.**
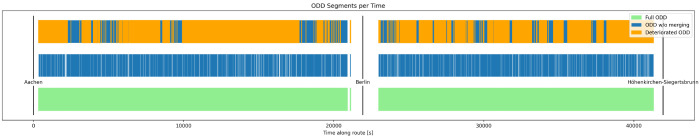
Possible ODD segments on a trip from Aachen to Höhenkirchen-Siegertsbrunn via Berlin based on a snapshot of https://download.geofabrik.de/europe/germany-latest.osm.pbf.

This initial assumption about the ODD is obviously too optimistic: Not only did we neglect other limiting factors like bad visibility, we neither investigated up to now if our specific function is actually able to handle all foreseeable situations regarding road geometry. One issue could be, for instance, that the initial version of the system cannot handle merge maneuvers into adjacent lanes. One of the ODD restrictions resulting from this fact is that the ODD excludes all situations along the motorway where the used lane ends and a merge into the neighbor lane becomes necessary. Such events are rather common on motorways, as can be seen in Figure 3. This severely restricts the system’s ODD and the system would have to be designed in a way that it can detect or know (e.g. from a feature-enriched on-board roadmaps) these situations ahead of time and alert the driver to take back control and do the lane merge manually.

A better metric on this version’s ODD would consider these situations. As explained at the end of the previous section, one way to do so would be to count the occurrences of such lane ending situations. In our off-board evaluation of the ODD we make use of OpenStreetMap, a roadmap that contains, among others, information about the number of lanes per direction. We can run a script over the map, based on the pre-selected route, and count the occurrences of the lane number reducing by one along the route. This can either directly serve as a metric (number of ODD gaps along the route, which is related to the user satisfaction with the AD function because the typical user doing side tasks will be annoyed by the need to take back control), or we can use this new information to adjust our initial ODD metric, which was percentage of the distance usable for the AD function. For the latter case, we would make realistic assumptions on the distance affected by the lane ending event, e.g. 1 km before and 200 m after a lane ending would be considered as outside the ODD (this is roughly the distance where human driver attention is needed).

There are more suitable metrics which show the impact of such ODD gaps and, thus, the potential for optimization: If the entire motorway sections were inside the ODD, this would result in two sections in the range of five to six hours. Taking the segmented ODD, which is partitioned by merging situations, around 250 take-overs would be required by lane ends. A maximum of only around 10 minutes per segment is achieved, with a mean duration of segments around 100 s (cf. I, Segmented ODD). From a technical perspective, this is calculated by checking for switches in the OpenStreetMap tag lanes and calculating the distance of coherent sections. Using the maximum allowed speed on that segment (min( maxspeed, 120 *km/h*)), the time within ODD is calculated.

As an improvement step to our AD function, let’s consider one of the enablers analyzed within Hi-Drive: cooperative merging. This enabler allows automated vehicles to deal with situations where traffic lanes merge, which were initially excluded from the ODD. After introducing this function that allows continuous automated driving without interruption each time when a lane ends, we can again use the full idealistic ODD, i.e. continuous automated driving for about 5 or 6 hours, or alternatively, 93 % of the trip time or 98 % of trip distance. Of course, this simple example neglects many other events that can equally constitute ODD gaps.

As a further example, consider the case of travelling on motorways with no speed limit. This is a case which often occurs in Germany but can pose a real challenge for an automated vehicle. Looking at cases where the automated vehicle wants to change the lane, a large area behind the vehicle needs to be monitored in order to assure the safety of the vehicle and its passengers when changing lanes. Therefore, segments of road which don’t have a speed limit must be considered as outside the ODD. This reduces the ODD to approx. 30 % of the total trip. This is calculated by setting the time spent on segments with a speed limit into relation with segments without a speed limit.

Again, the possibilities developed by the Hi-Drive project can partially mitigate this restriction and make the ODD bigger again. One option to do so is by introducing different levels of functionality, so that an ODD gap can be turned in a phase of "deteriorated ODD", in the meaning of outside the ODD of the full functionality but still inside the ODD of a degraded mode of the ADS, e.g. the ADS could still be active but the left-most lane will not be used. Allowing this degraded mode extends the original ODD and therefore reduces the number of take-over requests, but of course limits the number of lanes usable by the vehicle, thereby also reducing the average travel speed (there is more frequently the need to slow down for a slower predecessor vehicle as overtaking is not possible), which is another important metric along the ODD.

On the route planned in this paper, the ODD is deteriorated for large segments at a time. Similar to the previous calculations, this is done by setting the segments of different ODD conditions into relation to the complete trip. The following formulas apply:


$$ODD_{complete}=\frac{T_{complete}}{T_{complete}}=100\%,\;(1)$$

$$ODD_{w/o\;merge}=\frac{T_{w/o\;merge}}{T_{complete}},\;(2)$$

$$ODD_{w/o\;merge,\;w/\;speedlimit}=\frac{T_{w/o\;merge,\;w/\;speedlimit}}{T_{complete}},\;(3)$$

where *T _complete_* is the complete time of the trip on motorways calculated as described above. Similarly, *T _w/o merge_* is the time spent in areas without merging and *T _w/o merge, w/ speedlimit_* is the time spent in areas without merging and with speed limits.

It should be mentioned that the reality is much more complex as these examples: for instance, the maps shows for some road segments the presence of automatic sign bridges, which at some times display different speed limits but at other times don’t display anything at all and leave the allowed speed unlimited. This means that those segments are at some time part of the unrestricted ODD but at other times outside the ODD or part of the deteriorated ODD, respectively. Considering all those aspects in detail is beyond the scope of this article but can make a large difference in practice.

As a deteriorated ODD doesn’t necessarily mean that the driver has to take over control, the number of take-overs and the longest duration without take-over cannot be calculated without further information.

In any case, using different kinds of metrics, similar to the ones proposed here, enables the Hi-Drive project members to validate how much value they have added by making improvements to the automated driving function in order to extend its ODD and to reduce the need for human intervention.

## Conclusions

An ODD is based on a formalized ontology or taxonomy of all relevant entities in the "driving universe", and is effectively a partitioning of these entities into "what is in" and "what is out". Based on this approach, metrics can be defined using well-established approaches of set theory and basic arithmetic.

This paper discussed metrics for different properties of ODDS such as the "size" of an ODD or the quantification of how much of a map segment is within a given ODD. Metrics may also be used for the comparison of multiple ODDs to another or even to compare an ODD against other artifacts like scenarios. In this case one could determine which portion of a scenario lies within a given ODD or measure the coverage of an ODD by a set of test scenarios.

Starting from a very simple basic concept, some extensions have been proposed, for instance, to deal with continuous and unlimited value ranges or to compute one single number for the multidimensional aspects to be considered.

After demonstrating the approach by simple examples we applied metrics to a real world problem from the Hi-Drive project. Even with only the two elements taken into account, significant variance in the metrics was demonstrated.

The metrics demonstrated play an important role in the comparison of ODDs - either between different systems or evolutions of a single system. Harmonization and acceptance of these metrics along players in the field of driving automation will allow for easier and more understandable communication. Yet, a necessary precondition for the metrics to be applied is the agreement on an ontology which underlies set based operation required to calculate the metrics. Comparisons of differently defined metrics are not valid. Due to the high relevance of scenario-based testing it is recommended that future work focuses on the specifics of coverage metrics for scenarios with respect to a given ODD.

Future work within the Hi-Drive project, will focus on implementing enablers that close existing gaps in the ODD of automated systems as well as extend the ODD to new conditions, environments, and scenarios. The success of these efforts can then be evaluated using similar metrics to those presented in this paper. As a final step within Hi-Drive, the impacts of the enablers within the areas safety, efficiency and mobility will be analyzed, providing an estimation of their overall effectiveness.

Comparison and application of metrics and ODDs can only be successful if it is based on a shared understanding. Underlying all these efforts will be the ongoing integration with standardization activities, such as ASAM OpenODD. These efforts and others need to include a far wider range of stakeholders, including safety engineers, V&V engineers or road planners and public authorities.

## Data Availability

We in the application section, we used OpenStreetMap data provided as download by Geofabrik GmbH. Data can be accessed via https://download.geofabrik.de/europe/germany-latest.osm.pbf.

## Footnotes

^1^ Available at https://www.asam.net/standards/asam-openxontology/

^2^ We are aware that mathematicians have a very precise definition of metrics and distinguish related terms like measure, norm etc. However, our target audience are practitioners in engineering, who commonly use the term metric in a more relaxed way, e.g. as in "Quality Metrics" or "Software Test Metrics". So we assume that by saying "metrics" our approach will be more widely understood.

^3^ https://en.wikipedia.org/wiki/Jaccard_index

^4^ https://www.hi-drive.eu/

## References

[1] ISO 21448: Road vehicles. Safety of the intended functionality.Std.,2022. Reference Source

[2] ISO/TR 4804: 2020: Road vehicles — Safety and cybersecurity for automated driving systems — Design, verification and validation.Std.,2020. Reference Source

[3] UL 4600 Standard for Evaluation of Autonomous Products.Std.,2020. Reference Source

[4] Mazzega J Lipinski D Eberle U : Pegasus method.Tech. Rep., 05,2019. Reference Source

[5] Tonk A Boussif A Beugin J : Towards a specified operational design domain for a safe remote driving of trains.In: *ESREL 2021, 31st European Safety And Reliability Conference.* Angers, France,2021. Reference Source

[6] SAE J3016: Taxonomy and Definitions for Terms Related to Driving Automation Systems for On-Road Motor Vehicles.Std.,2021. Reference Source

[7] BSI PAS 1883: Operational design domain (ODD) taxonomy for an automated driving system (ADS). Specification.Std.,2020. Reference Source

[8] ASAM e.V: ASAM OpenODD concept paper. 2021. Reference Source

[9] ASAM e.V: ASAM OpenXOntology concept paper. 2022. Reference Source

[10] Kaiser B : Realizing metrics for odds and simulation scenarios.In: ASAM Technical Seminar 2021,2021. Reference Source

[11] Gyllenhammar M Johansson R Warg F : Towards an operational design domain that supports the safety argumentation of an automated driving system.In: *10th European Congress on Embedded Real Time Software and Systems (ERTS 2020).* TOULOUSE, France, Jan.2020. Reference Source

[12] Whiteside I : Ontologies and odds at five.Presentation at Ideation Workshop ASAM OpenODD, Apr 23,2020; 2020. Reference Source

[13] McGuinness DL Van Harmelen F : Owl web ontology language overview. *W3C recommendation.* 2004;10(10):2004. Reference Source

[14] Horrocks I Patel-Schneider PF Boley H : Swrl: A semantic web rule language combining owl and ruleml. *W3C Member submission.* 2004;21(79):1–31. Reference Source

[15] Thorn E Kimmel S Chaka M : Framework for automated driving system testable cases and scenarios.Tech. Rep.,2018. Reference Source

[16] Koopman P Fratrik F : How many operational design domains, objects, and events?In: SafeAI@AAAI,2019. Reference Source

[17] ISO 26262: Road vehicles - Functional safety - Part 3: Concept Phase.Std.,2018. Reference Source

[18] Certified tester foundation level syllabus. 2011. Reference Source

[19] ISO 26262: Road vehicles - Functional safety - Part 6: Product development at the software level.Std.,2018. Reference Source

