# Supplementary material for: Towards the definition of metrics for the assessment of operational design domains
Source: Open Res Eur. 2023 Sep 11;3:146. [Version 1] doi: 10.12688/openreseurope.16036.1 (PMC10585198; doi:10.12688/openreseurope.16036.1)
Supplement: Peer review history

# Reviewer report: Reviewer response for version 1

**DOI:** 10.21956/openreseurope.17314.r35061

**Published:** 2023-10-18

**Authors:** Rasmus Adler

**Affiliations:** 1 Fraunhofer IESE, Kaiserslautern, Germany

The paper discusses a very important topic. There are various notions and uses cases related to the term ODD. The paper contributes to clarity about some notions and use cases. Formalizing the ODD with a set-theoretic approach is reasonable.

 I was missing a little bit of discussion about the question "What is a driving situation?" Situations that are not distinguishable for humans can distinguished by vehicles and vice versa. In other words, humans see whether condition like rain or snow but the sensors only measure some phenomena. How to deal with the semantic gap between the natural language and that what is actually measured and causes the constraints leading to the ODD?  

 Minor comments:

 - "A set-theoretic approach to odd specifications"

    -> ODD (in capitals)

 - sets, depending ONThe attribute we are talking about

   -> typo

 All in all its an interesting paper and worth to be published

Is the rationale for developing the new method (or application) clearly explained?

Yes

Is the description of the method technically sound?

Yes

Are the conclusions about the method and its performance adequately supported by the findings presented in the article?

Yes

If any results are presented, are all the source data underlying the results available to ensure full reproducibility?

Yes

Are sufficient details provided to allow replication of the method development and its use by others?

Yes

Reviewer Expertise:

Safety of autonomous systems. My reserach interest is not limited to automated driving and road vehicles. I have an overview about the discussion in research about the ODD. I already published in this field but i am not expert knowing all formats, languages, standardization activities and so on.

I confirm that I have read this submission and believe that I have an appropriate level of expertise to confirm that it is of an acceptable scientific standard.

# Reviewer report: Reviewer response for version 1

**DOI:** 10.21956/openreseurope.17314.r35060

**Published:** 2023-10-17

**Authors:** Steven E. Shladover

**Affiliations:** 1 California PATH Program, Institute of Transportation Studies, University of California Berkeley, Berkeley, California, USA

This paper raises some important points about the operational design domain (ODD) of Automated Driving Systems (ADS) that are often overlooked and misunderstood.  It is particularly useful in explaining the representation of the ODD in terms of a multi-dimensional hyperspace, to expand beyond the common oversimplification of defining it strictly in geographical terms. 

 However, its explanation of that multi-dimensional hyperspace is still somewhat over-simplified, based on an implicit assumption that those dimensions can be defined independently of each other.  Unfortunately, ODDs for real ADS are significantly more complicated than that, with couplings among the dimensions that lead to the need for non-convex hyperspace descriptions.  For example, reduced visibility caused by fog may preclude the ADS from operating in roadway curves with radii less than some threshold value or on high-speed highway segments that prohibit driving below a certain minimum speed.  This coupling can extend to significantly more dimensions – a good real-world example would be a system that is blinded by bright sunlight at low sun angles, which would need to exclude from its ODD westbound travel on certain roads at the specific times of day and seasons of the year, during clear weather conditions, when the setting sun prevents its sensors from detecting traffic signals and signs.

 The assessment of ODDs is important for a variety of reasons associated with determining the suitability of an ADS for serving specific transportation needs or use cases, or for determining whether a set of test scenarios is sufficient to assess the safety of an ADS.  However, this paper seems to devote excessive attention to the “size” of the ODD as its target, which raises a couple of concerns.  First, this places too much emphasis on the geographic dimension of the ODD, without giving sufficient attention to the range of environmental and traffic conditions that are just as important.  Secondly, comparisons of the “size” of the ODDs of different ADS designs does not look like a particularly useful exercise.  The important evaluation criterion is the match between the ODD and the requirements for the intended use case of the ADS.  If that use case is local urban package delivery, the ODD only needs to include the streets within the intended service area of the ADS in a specific city, and it may not need to include all traffic, weather and lighting conditions.  If the use case is long-haul trucking the ODD may only need to include limited-access motorways connecting distribution centers, and only under light traffic conditions and night-time darkness.  The limited scope of these ODDs is not necessarily a disadvantage.

 The paper mentions the need to base the ODD assessments on a common classification scheme, but that would only be relevant if one were trying to do an A-B comparison of different ADS, which does not look like a particularly useful activity (unless somebody wants to make an over-simplified marketing claim that his ODD is bigger than his competitor’s).  The more useful application of ODD assessment would be determining the adequacy of a suite of evaluation scenarios for assessing the safety of each ADS within its ODD, which does not require a common classification scheme.  This is a concern because at the current early stage in the development of ADS none of the proposed ODD classification schemes appears to be adequate to serve the purpose.  The ODD classifications need to be comprehensive (spanning ALL of the variables that affect the ability of an ADS to perform the dynamic driving task (DDT)), non-overlapping (clearly segregating the categories of variables to avoid double-counting) and conceptually orthogonal (enabling the representation of coupling among the variables that may interact with each other to limit ADS performance of the DDT).  

 The ODD description of an ADS needs to include ALL factors external to the vehicle that may constrain the ability of the ADS to perform the DDT, otherwise it is incomplete and therefore faulty.  The discussion in the first column of page 9 of the paper implies that the ODD specification would not include all of these factors, and includes the strange statement that “Regarding traffic participants, it is only mentioned which classes of them can be present, and what speed ranges can be expected.”  If any other aspects of the other traffic participants affect the ability of the ADS to perform the DDT (if it cannot recognize and respond appropriately to dark-colored vehicles, or emergency vehicles, or roadworks zones, or micro-mobility device users) those must be included in the ODD description.  An ODD description that only covers road type and geometry and weather conditions but omits consideration of road surface conditions and any surrounding factors that affect DDT performance would be seriously deficient.   Because the environmental and traffic condition aspects of the ODD are inherently stochastic and unpredictable and have coupled effects on DDT performance, it is not feasible to use them to answer the questions that the authors have raised in the first column of page 9.

 Narrower concerns:

- The abstract suggests using the ODD metrics to judge the maturity of a system with a certain ODD, but this seems dubious because the complexity of the ODD itself is the more relevant indicator of ADS maturity (given that the ADS needs to be capable of performing the DDT throughout its ODD, regardless of how simple or complicated that ODD may be).  Contrary to the statement in the Introduction, the ODD does not determine the capabilities of an ADS, because those capabilities must, by definition, cover performance of the entire DDT.  The example cited in the section of the paper called “from attribute-level metrics to ODD-level metrics” (what percentage of all typical driving tasks can be performed in automated mode) is therefore incorrect, because that percentage needs to be 100% within the ODD for any ADS.
- “Non-deteriorated DDT” is used as a column heading in Table 1 but is not defined until significantly later in the paper, which makes it confusing to the reader.

Is the rationale for developing the new method (or application) clearly explained?

Partly

Is the description of the method technically sound?

Yes

Are the conclusions about the method and its performance adequately supported by the findings presented in the article?

Partly

If any results are presented, are all the source data underlying the results available to ensure full reproducibility?

No source data required

Are sufficient details provided to allow replication of the method development and its use by others?

Partly

Reviewer Expertise:

My research covers many aspects of intelligent transport systems, with a particular emphasis on automated driving systems, including the development and testing of the technology, evaluating its impacts on the transport system, and investigating its policy implications. I have been working on automated driving systems for 50 years, beginning with my MIT masters thesis.

I confirm that I have read this submission and believe that I have an appropriate level of expertise to confirm that it is of an acceptable scientific standard, however I have significant reservations, as outlined above.

# Reviewer report: Reviewer response for version 1

**DOI:** 10.21956/openreseurope.17314.r35056

**Published:** 2023-09-12

**Authors:** Ulrich Eberle

**Affiliations:** 1 Stellantis NV/ Opel Automobile GmbH, Rüsselsheim am Main, Germany

The term and concept of Operational Design Domain is crucial for the implementation of safe Automated Driving over a wide range of automation levels, covering both ADAS and Automated Driving L3/L4/L5.

 ASAM standards of the OpenX family (initially developed in PEGASUS family projects) play an important role for the industry-wide application of scenario-based development, testing and validation.

 Ontologies are a valuable tool to describe, structure and understand the respective parameter space which leads to the so-called Open-World-Challenge.

 The general concept and methodological approach of the piece and the related activities are reasonably lined out.

 Minor points:

- In the Introduction, the Code-of-Practice as developed within  L3Pilot should be mentioned as "guiding star" for the development and validation process where ODD plays an instrumental role.
- section headline should read "Deriving metrics for **ODDs**" instead of "... **odds**".

Is the rationale for developing the new method (or application) clearly explained?

Yes

Is the description of the method technically sound?

Yes

Are the conclusions about the method and its performance adequately supported by the findings presented in the article?

Yes

If any results are presented, are all the source data underlying the results available to ensure full reproducibility?

Yes

Are sufficient details provided to allow replication of the method development and its use by others?

Yes

Reviewer Expertise:

Automated Driving, Novel simulative methodologies for development and validation of automated driving.

I confirm that I have read this submission and believe that I have an appropriate level of expertise to confirm that it is of an acceptable scientific standard.
